# Genetic Diversity and Population Structure of the Common Carp (*Cyprinus carpio*) in Albania Revealed by Microsatellite Markers

**DOI:** 10.3390/life16091524

**Published:** 2026-09-14

**Authors:** Anila Hoda, Chiara Locci, Ilenia Azzena, Ilaria Deplano, Maria Perra, Giada Lostia, Valbona Kolaneci, Arjan Demiri, Fabio Scarpa, Daria Sanna, Marco Casu

**Affiliations:** 1 Academy of Sciences of Albania, Shëtitorja Murat Toptani, 1000 Tirana, Albania; anila.hoda@akad.gov.al; 2 Department of Veterinary Medicine, University of Sassari, Via Vienna 2, 07100 Sassari, Italy; clocci1@uniss.it (C.L.); iazzena@uniss.it (I.A.); 3 Department of Biomedical Sciences, University of Sassari, Viale San Pietro 43b, 07100 Sassari, Italy; i.deplano@phd.uniss.it (I.D.); m.perra9@phd.uniss.it (M.P.); giadalostia02@yahoo.com (G.L.); fscarpa@uniss.it (F.S.); 4 Department of Aquaculture and Fishery, Agricultural University of Tirana, Koder Kamëz, 1029 Tirana, Albania; vkolaneci@ubt.edu.al; 5 Directory of Fishery and Aquaculture Services, Blvd Deshmoret e Kombit, Nr. 2, 1001 Tirana, Albania; arjan.demiri@dshpa.gov.al; 6 Department of Innovation, University of Sassari, Via Porto Romano 8, 07026 Olbia, Italy; marcasu@uniss.it

**Keywords:** microsatellites, genetic variability, stocking programmes, genetic admixture, inland fisheries

## Abstract

Understanding genetic diversity and population structure is essential for the sustainable management of freshwater fish, particularly in systems influenced by long-term stocking. In this context, the genetic status of Albanian common carp (*Cyprinus carpio*) remains poorly understood. Here, 125 individuals from five populations representing natural lakes (Ohrid and Shkodra), semi-managed freshwater systems (Prespa and Belshi), and a hatchery stock (Tapiza) were genotyped at 12 polymorphic microsatellite loci. High genetic diversity was detected across all populations, with low genetic differentiation and relevant estimates of gene flow. Bayesian clustering identified two main genetic groups, with natural lake populations predominantly represented by a single genetic lineage and managed systems showing higher admixture. Multivariate and phylogenetic analyses supported a close genetic relationship between Ohrid and Shkodra natural lake populations. These patterns may reflect historical stocking and translocation programmes, potentially involving genetically similar or shared broodstock sources, which may have contributed to the observed genetic similarity among populations while maintaining high levels of within-population genetic diversity. The more homogeneous genetic composition of natural lake populations may also reflect local environmental conditions that influenced the distribution of allelic variants among populations. Overall, this study provides a comprehensive genetic assessment of Albanian common carp and establishes a baseline for future sustainable management plans.

## 1. Introduction

Genetic diversity is an essential component of biodiversity and plays a central role in the long-term persistence of natural populations [1,2]. It enables populations to adapt to changing environmental conditions and emerging pathogens [1,3,4]. Moreover, reduced genetic diversity may limit populations’ adaptive potential in response to stressors and increase their vulnerability to environmental pressures, thereby reducing their fitness [1,4]. The processes shaping genetic diversity are strongly influenced by species ecology and landscape characteristics, which determine dispersal dynamics, connectivity, and genetic exchange among populations [5]. Consequently, ecosystems in which connectivity is naturally constrained or easily disrupted provide particularly valuable models for investigating the impacts of environmental changes on population genetic structure [5].

This phenomenon is particularly evident in freshwater ecosystems, where the dendritic structure of river networks naturally constrains dispersal and gene flow, making these systems especially suitable for evaluating genetic responses to changing environmental conditions and habitat discontinuity [5,6]. Furthermore, freshwater ecosystems are exposed to human disturbances, such as habitat alteration, fragmentation, overexploitation, and fish stocking, which have modified natural patterns of connectivity, often leading to changes in population structure and the erosion of locally adapted genetic variation [6]. For this reason, assessing the genetic diversity and population structure of freshwater animals is crucial for the effective management, conservation, and sustainable use of aquatic resources and represents a primary objective of conservation genetics and fisheries management.

The common carp, *Cyprinus carpio* Linnaeus, 1758, has a remarkably broad geographic distribution and considerable economic relevance among freshwater fish species worldwide and has been extensively exploited in fisheries and aquaculture for centuries [7]. Native to Eurasia, the species has undergone a long history of domestication, translocation, and artificial propagation, resulting in a broad geographic distribution that extends far beyond its native range. These processes have strongly influenced the genetic composition of both wild and stocked populations, generating complex genetic patterns shaped by both natural evolutionary processes and human-mediated introductions of different genetic lineages [8]. Furthermore, over the course of their domestication, hatchery-derived and domesticated carp have been repeatedly translocated and stocked into rivers and lakes, where they have hybridized with native wild populations. As a result, present-day populations often represent a mosaic of wild and domesticated genetic lineages, reflecting multiple historical introductions, admixture events, and introgression events that have increased the complexity of their genetic structure [8,9]. This is particularly evident in many regions, including the Mediterranean freshwaters, where repeated stocking practices and the translocation of hatchery-reared individuals have promoted gene flow and admixture among populations, often reducing genetic differentiation [9].

In the Western Balkans, *Cyprinus carpio* plays a central role in inland fisheries and aquaculture [10]. The region encompasses several ecologically significant freshwater ecosystems, notably Lakes Ohrid, Prespa, and Shkodra, which are recognized as biodiversity hotspots that support highly diverse fish communities. However, increasing anthropogenic pressures have altered patterns of population connectivity, contributing to changes in the genetic structure of freshwater fishes [11,12]. In this context, Albania comprises a heterogeneous mosaic of aquatic environments, ranging from natural lakes to semi-managed systems alongside carp hatchery stocks. Together, these systems provide a valuable natural framework for investigating how natural evolutionary processes and human activities interact to shape the genetic variation and structuring within common carp populations [13].

Given its worldwide distribution, largely resulting from centuries of human-mediated translocations beyond its native range, and its high economic importance for fisheries and aquaculture, *Cyprinus* carpio has been extensively investigated from a population genetics perspective (e.g., [7,8,14,15,16,17,18,19,20]). Much of this research has relied on microsatellite markers and has revealed considerable genetic variation across common carp populations, while patterns of population structure often vary according to the intensity of stocking and management practices. Hatchery stocks, in particular, frequently exhibit signatures of admixture, altered genetic composition, and reduced diversity (e.g., [16,21,22]).

Despite the extensive genetic research conducted on common carp worldwide, information regarding the genetic variability and population structure of Albanian populations remains limited (e.g., [23,24]). Previous investigations primarily relied on populations from major natural lakes, including Ohrid and Shkodra, and the semi-managed Prespa system, as well as hatchery stocks such as Tapiza and Klos, providing preliminary insights into patterns of genetic differentiation among populations (e.g., [23,24]). However, these investigations relied on a restricted panel of microsatellite loci, which may have constrained the resolution of inferred genetic diversity and population structure [23,24]. Furthermore, genetic information from smaller and semi-managed Albanian freshwater systems remains scarce, limiting our knowledge of how populations are genetically structured and interconnected in these often fragile ecosystems [25].

Using 12 polymorphic microsatellite markers, the present study aims to assess genetic variation, population-level structuring, and potential gene flow among five Albanian carp populations from the natural lakes Ohrid and Shkodra, the semi-managed basins of Prespa and Belshi, and the hatchery stock of Tapiza to address the aforementioned knowledge gaps. Although genome-wide approaches provide powerful alternatives, microsatellites remain a reliable and cost-effective tool for evaluating genetic diversity, population differentiation, and gene flow [26]. Hence, microsatellite markers are one of the most effective molecular tools for population genetic investigations owing to their high degree of polymorphism and strong resolution power, enabling fine-scale assessments of genetic variation within and among populations [27].

By expanding both the geographic coverage and the number of microsatellite loci employed, compared with previous studies, this study also aims to contribute valuable information to support the sustainable management and long-term monitoring of *Cyprinus carpio* in Albanian freshwater ecosystems.

## 2. Materials and Methods

### 2.1. Sample Collection

**Figure 1:**
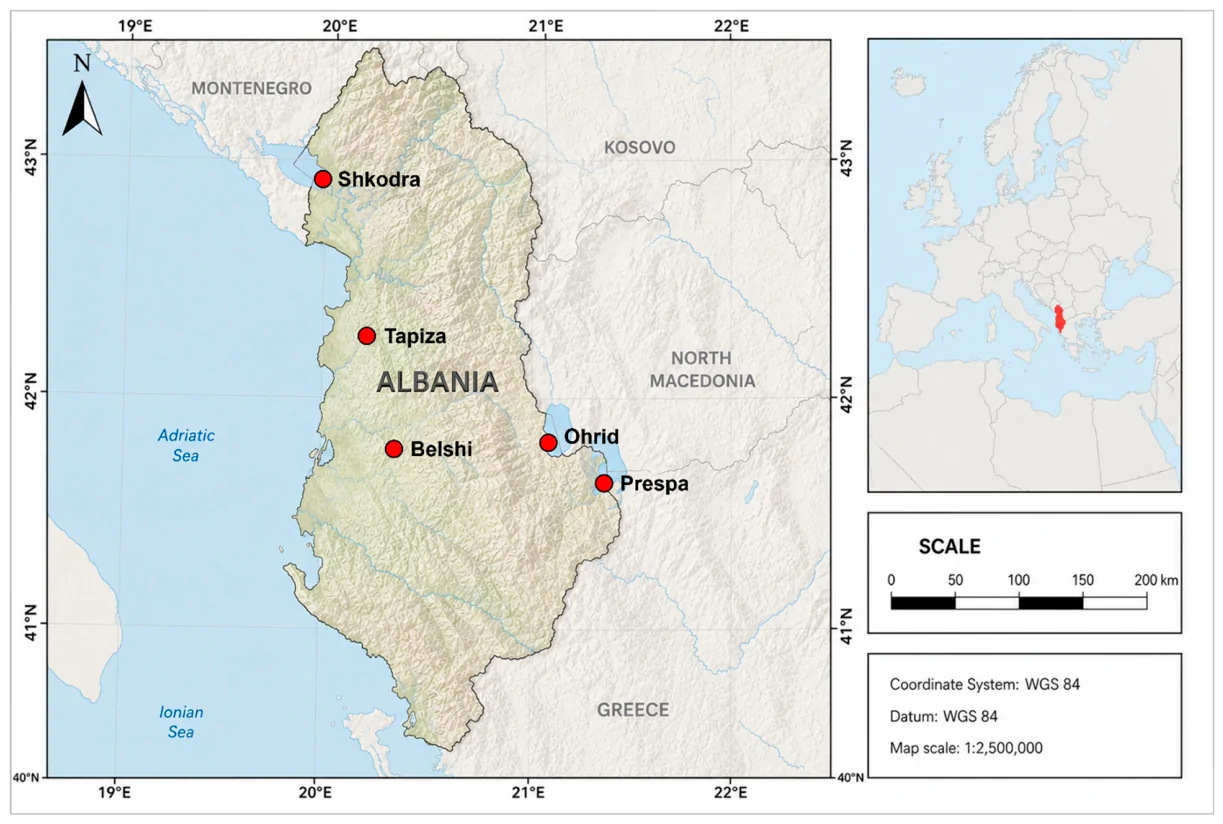
Sampling locations of Albanian *Cyprinus carpio* populations from five different sampling sites.

A total of 125 individuals of common carp (*Cyprinus carpio*) were sampled from five distinct populations in Albania: Ohrid, Prespa, Shkodra, Belshi (Dega) Lake, and the Tapiza hatchery (see Figure 1 for sampling location details). From each population, 25 individuals were randomly selected to ensure representative sampling. For the transboundary lakes Shkodra, Ohrid, and Prespa, samples were obtained exclusively from fish caught within the Albanian sectors of the lakes. The specimens originated from fishing activities conducted at different fishing points within each Albanian lake sector, thereby providing spatial coverage within the sampled areas. Lake in Belshi (Dega) is located entirely within central Albania, and samples were obtained from routine fishing activities conducted within the lake. At the Tapiza hatchery, the individuals were sampled from three different rearing ponds to avoid restricting the hatchery sample to a single pond.

Fish tissue sampling was carried out with the assistance of local fishermen and representatives of fisheries management organizations (OMPs), who facilitated access to sampling sites and fishing activities.

All specimens originated exclusively from fish captured during routine fishing activities and intended for personal consumption or commercial sale. From the available catches, 25 individuals per locality were selected for genetic analysis.

The present study did not involve the deliberate sacrifice of any fish. Sampling was conducted after fishing operations, ensuring that all biological material was derived from already deceased individuals. Fin clips were excised from the caudal fin of each individual after capture and conserved in 1.5 mL tubes containing 96% ethanol. The samples were subsequently transferred to the laboratory and maintained at −20 °C prior to DNA extraction.

### 2.2. DNA Isolation

Genomic DNA was extracted from caudal fin tissue with the NucleoSpin Tissue Kit (MACHEREY-NAGEL GmbH and Co. KG, Duren, Germany), following the protocol provided by the manufacturer. DNA quantity and purity were assessed with a Nanodrop™ Lite Spectrophotometer (Thermo Scientific; Waltham, MA, USA), with DNA extracts showing a mean concentration of approximately 100 ng/μL.

### 2.3. Microsatellite Markers

Prior to microsatellite genotyping, DNA quality was verified through PCR amplification of a fragment of the mitochondrial Control Region employing the primers L19CR (forward) (5′-CCACTAGCTCCCAAAGCTA-3′; [28]) and K-Rev CR (reverse) (5′-CAGGACCAAGCTTTTGTGCTTACG-3′; [29]). Successful amplification was considered indicative of adequate DNA integrity and suitability for subsequent microsatellite analyses.

Microsatellite loci were selected from a panel of 30 loci developed by Li et al. [20] (https://doi.org/10.1016/S1673-8527(07)60111-8), originally designed for common carp. From this initial panel, a subset of 12 highly polymorphic loci was selected (044, 058, 896, 049, 055, 380, 392, 398, 806, 817, 873, and 338; see Table 1 for details) based on the levels of genetic variability reported by Li et al. [20]. Specifically, the 12 loci analysed in the present study showed the highest overall values of effective number of alleles (A_e_), observed heterozygosity (H_O_), and polymorphism information content (PIC) among the 30 loci evaluated in the original study. PCR conditions were optimised through simplex reactions and subsequently organised into three multiplex assays and one simplex assay according to their annealing temperatures (Table 1). The final amplification scheme included: Multiplex 1 (loci 044, 058, 896, and 398; annealing temperature 53 °C), Multiplex 2 (loci 049, 055, 380, and 392; annealing temperature 54 °C), Multiplex 3 (loci 806, 817, and 873; annealing temperature 48 °C), and one simplex reaction for locus 338 (annealing temperature 51 °C).

PCR amplifications were set up in a final volume of 10 μL comprising approximately 20 ng of genomic DNA, 0.4 μM of each locus-specific primer, and one PuReTaq Ready-To-Go PCR bead (GE Healthcare, Wauwatosa, WI, USA). Each reaction provided 2.5 U of PuReTaq DNA polymerase, 200 μM of each dNTP, and 1.5 mM MgCl_2_. Thermal cycling was conducted on a GeneAmp PCR System 9700 (Applied Biosystems, Waltham, MA, USA), beginning with denaturation at 94 °C for 4 min. This was followed by 35 cycles consisting of 30 s at 94 °C, 10 s at the locus-specific annealing temperature, and 30 s at 72 °C. Reactions were then subjected to a 10 min final extension at 72 °C and maintained at 4 °C. Amplification success was assessed by electrophoresis on 2% agarose gels stained with GelRed Nucleic Acid Stain (Biotium Inc., Fremont, CA, USA). PCR products were subsequently diluted 1:10 with Milli-Q water and sent to Macrogen Europe (Milan, Italy) for microsatellite genotyping.

Allele scoring and binning were performed using Geneious 2026.0.2 (https://www.geneious.com, access date: 28 April 2026). Fragment analysis was conducted considering the expected allele size range reported by Li et al. [20], spanning from 125 to 414 bp across all loci included in the study.

### 2.4. Statistical and Population Genetic Analyses

MICRO-CHECKER v. 2.2.3 [30] was employed to screen the dataset for genotyping errors, including large allele dropout and scoring errors due to stuttering. In addition, FreeNA [31] (https://umr-cbgp.fr/en/logiciel/freena-2/?utm_source=chatgpt.com, access date: 25 April 2026) was employed to estimate the frequency of null alleles (NA) across all loci, which ranged from 0.000 to 0.151.

Genetic diversity parameters, comprising total number of alleles per locus (TNA), allelic frequencies, private alleles (PA), observed (H_O_) and expected heterozygosity (H_E_), were assessed using GenAlEx v. 6.5 [32], while polymorphic information content (PIC) was estimated with CERVUS v. 3.0.7 [33]. Departures from Hardy–Weinberg equilibrium (HWE) were assessed separately for each of the 60 population × locus combinations using the exact probability test implemented in GENEPOP v. 4.7.5 [34]. Exact probabilities were estimated using a Markov chain with 10,000 dememorization steps, 1000 batches, and 10,000 iterations per batch. Results were initially evaluated at the nominal significance level of (*p* < 0.05). To account for the 60 population × locus comparisons (five populations × 12 loci), a Bonferroni-adjusted threshold of (*p* < 0.00083) (0.05/60) was subsequently applied. GENEPOP also combined the population-specific probabilities for each locus across the five populations using Fisher’s method. These locus-level combined probabilities were reported separately at their nominal significance levels. Genetic differentiation and inbreeding were assessed using Weir and Cockerham’s (1984) F-statistics (F_IS_, F_ST_, F_IT_) [35] in FSTAT 2.9.4 [36], with significance evaluated by permutation tests (1000 iterations) and robustness assessed via jackknife and bootstrap resampling. Pairwise F_ST_ values were calculated in Genetix [37], and gene flow (Nm) was estimated following Wright [38].

Nei’s standard genetic distances [39] were used to generate a neighbor-joining dendrogram [40], with 1000 bootstrap replicates implemented in POPULATION v. 1.2.28 (see [7] and the references therein for details) to explore relationships among the populations. In addition, an individual-level neighbour-joining tree was generated to assess genetic relationships among sampled individuals. The resulting trees were visualized in MEGA v. 11.0.13 [41].

Potential departures from neutral expectations were evaluated using the Bayesian FST-outlier approach implemented in BayeScan v. 2.1 [42]. The analysis included all 12 codominant microsatellite loci and the five sampled populations. Twenty pilot runs of 5000 iterations each were performed, followed by an additional burn-in of 50,000 iterations. Posterior estimates were based on 5000 samples recorded at a thinning interval of 10. Prior odds of 10 were assigned in favour of the neutral model. Candidate outlier loci were identified using the estimated q-values, with a false discovery rate threshold of (q < 0.05). Because the neutral background was estimated from only 12 microsatellite loci, this analysis was treated as an exploratory screening for candidate outliers.

Population genetic structure was investigated using two complementary approaches. First, an analysis of molecular variance (AMOVA) was performed in GenAlEx [32] to partition genetic variation among sampling populations and among individuals within populations. The analysis included all 125 individuals genotyped at 12 microsatellite loci. The statistical significance of the variance components and the global fixation index (F_ST_) was evaluated using 999 standard permutations across the complete dataset. Pairwise F_ST_ values between populations were also calculated in GenAlEx, with their statistical significance assessed using 999 permutations.

Bayesian clustering was performed in STRUCTURE v. 2.2 [43] using an admixture model with correlated allele frequencies. Analyses were conducted for K = 1–6 with 10 independent runs per K, using a burn-in of 100,000 iterations followed by 200,000 MCMC iterations. The most plausible number and hierarchical pattern of clusters were evaluated using complementary criteria, including mean lnP(D), the ΔK method of Evanno et al. [44], and the MedMedK, MedMeaK, MaxMedK, and MaxMeaK estimators implemented in StructureSelector [45], using membership thresholds of 0.5–0.8. Replicate runs were aligned and summarized in CLUMPAK [46] [https://lmme.ac.cn/StructureSelector/, access date: 26 August 2026] to identify major clustering modes and generate consensus individual-membership bar plots. The analysis was additionally repeated after excluding HLJ338 to assess the sensitivity of the inferred structure to the locus showing evidence of potential null alleles.

Multivariate analyses, including principal coordinate analysis (PCoA) in GenAlEx [32] and factorial correspondence analysis (FCA) in Genetix v. 4.0.5.2 [37], were used to visualize genetic relationships. Bottleneck effects were assessed using Bottleneck v.1.2.0.2 [47]. Departures from mutation–drift equilibrium were evaluated under the infinite allele model (IAM), stepwise mutation model (SMM), and two-phase mutation model (TPM) [48]. For the TPM, 70% single-step mutations and a variance of 30 were specified, and equilibrium heterozygosity was estimated using 1000 simulations. Sign tests and two-tailed Wilcoxon signed-rank tests for heterozygosity excess or deficiency were applied. Allele-frequency distributions were additionally examined using the mode-shift indicator [49], with a normal L-shaped distribution interpreted as evidence against a recent severe bottleneck. Contemporary effective population size (Ne) was estimated separately for each population using NeEstimator v. 2.1 [50] assuming random mating and applying a minimum allele-frequency threshold of 0.02. Parametric 95% confidence intervals were reported. The primary estimates were obtained using the complete 12-locus panel. To evaluate the robustness of the LD-based Ne estimates, sensitivity analyses were conducted using reduced marker panels. Loci showing evidence of possible null alleles and one locus from each pair displaying significant linkage disequilibrium after Bonferroni correction were sequentially excluded. The resulting estimates were compared with those obtained from the complete 12-locus panel.

Pairwise genotypic linkage disequilibrium (LD) among the 12 microsatellite loci was tested separately within each population using exact tests implemented in GENEPOP [34]. Exact probabilities were estimated using a Markov chain with 10,000 dememorization steps, 1000 batches, and 10,000 iterations per batch. Because 66 locus pairs were evaluated in each of the five populations, a total of 330 population × locus-pair comparisons were considered. Results were initially evaluated at the nominal significance level of (*p* < 0.05), followed by application of a Bonferroni-adjusted threshold of (*p* < 0.000152) (0.05/330). GENEPOP also combined the population-specific probabilities for each of the 66 locus pairs across populations using Fisher’s method. For these combined tests, the corresponding Bonferroni-adjusted threshold was (*p* < 0.000758) (0.05/66).

Individual self-assignment and first-generation migrant detection were performed using GeneClass2 v. 2.0 [51]. Genotype likelihoods were calculated using the Bayesian criterion of Rannala and Mountain (1997) [52]. For self-assignment, each individual was assigned to the reference population yielding the highest multilocus genotype likelihood. No probability threshold was applied to the self-assignment classification, and the procedure was not leave-one-out cross-validated. First-generation migrant detection was performed using the Monte Carlo resampling algorithm of Paetkau et al. [53], with 10,000 simulated individuals and a Type I error threshold of α = 0.01. The L_home_ statistic, representing the likelihood of an individual’s genotype in its sampled population, was used as the primary criterion, whereas L_home_/L_max_ was examined as a complementary criterion. No additional correction for multiple testing was applied; consequently, individuals meeting the probability threshold were treated as candidate first-generation migrants rather than confirmed migrants. To assess the potential influence of null alleles at HLJ338, the self-assignment and migrant-detection analyses were repeated after excluding this locus.

## 3. Results

### 3.1. Genetic Variability of Microsatellite Markers

Screening with Micro-Checker detected neither stuttering nor large allele dropout at any of the analysed loci. In contrast, HLJ338 showed indications of null alleles in all populations except the Tapiza hatchery, whereas no other locus displayed a consistent null-allele signal across populations.

Frequencies of null alleles estimated using FreeNA were generally low (0.000–0.151) (Table S1). Evidence of null alleles above the commonly accepted threshold (r > 0.10) was detected only at locus HLJ338 in several populations, supporting the Micro-Checker results. These findings suggest a locus-specific heterozygote deficit rather than a widespread genotyping error; therefore, HLJ338 was retained but interpreted with caution in subsequent analyses.

The analysis of 12 microsatellite loci revealed a high level of genetic diversity across the studied populations (see Table 2 for details). The total number of alleles per locus (TNA) ranged from 4 (HLJ817) to 25 (HLJ806), with an average of 17.67 alleles per locus. The effective number of alleles (Ae) varied between 2.047 and 7.794, with a mean value of 5.387. Shannon’s information index (I) ranged from 0.738 to 2.275, indicating substantial allelic variability among loci.

Observed heterozygosity (H_O_) was consistently high, ranging from 0.709 (HLJ338) to 1.000 (HLJ044), and exceeded expected heterozygosity (H_E_) at the majority of loci (Table 1). The average H_O_ (0.942) was notably higher than H_E_ (0.778), indicating an overall excess of heterozygotes. The polymorphic information content (PIC) values ranged from 0.393 (HLJ817) to 0.887 (HLJ806), with a mean of 0.788, confirming that most of loci were highly informative. Allelic richness (AR) varied from 2.422 to 14.742, with an average value of 10.648.

Fisher’s combined tests across the five populations indicated nominally significant locus level departures from Hardy–Weinberg equilibrium at several loci as shown in Table 2).

F-statistics analysis showed predominantly negative F_IS_ values across loci (mean F_IS_ = −0.192; Table 2), indicating a general excess of heterozygotes and no overall heterozygote deficit of the type expected under inbreeding in the analysed samples. The only exception was locus HLJ338, which exhibited a positive F_IS_ value (0.178), suggesting a heterozygote deficit at this locus. The overall F_IT_ value was also negative (−0.150), supporting the observed heterozygosity excess at the global level (see Table 2).

Populations exhibited limited but significant genetic differentiation, with F_ST_ values ranging from 0.000 (HLJ817) to 0.063 (HLJ380), and an average value of 0.035 (*p* < 0.01). All loci showed highly significant differentiation (*p* < 0.001), except HLJ817, which was not significant (Table 2). These results indicate weak but significant population structure despite the high genetic diversity observed.

### 3.2. Population Genetic Diversity

Genetic diversity was from moderate to high across the five carp populations (Table 3). The mean number of alleles (MNA) ranged from 8.58 in Tapiza to 11.50 in Shkodra, indicating substantial allelic richness. Similarly, the effective number of alleles (Ae) was highest in Shkodra (6.71) and lowest in Tapiza (4.65), suggesting slightly greater genetic variability in natural populations compared to the hatchery stock. Shannon’s information index (I) followed the same pattern, with the highest value observed in Shkodra (2.031).

Hardy–Weinberg equilibrium was evaluated separately for each of the 60 population × locus combinations (Table 3). At the nominal significance level (*p* < 0.05), deviations from HWE were detected in 33 combinations. After applying the Bonferroni-adjusted threshold (*p* < 0.00083), 12 combinations remained significant. HLJ817 deviated significantly from HWE in all five populations, whereas the remaining significant deviations were population-specific.

Observed heterozygosity (H_O_) was consistently high (0.909–0.993) and exceeded expected heterozygosity (H_E_), resulting in negative F_IS_ values across all populations (mean F_IS_ = −0.192). This pattern indicates an excess of heterozygotes relative to Hardy–Weinberg expectations and provides no evidence of the heterozygote deficit typically associated with inbreeding. The exploratory BayeScan analysis detected no significant F_ST_-outlier loci after controlling the false discovery rate at 5%. The q-values ranged from 0.829 for HLJ049 to 0.924 for HLJ873 and were therefore substantially higher than the (q < 0.05) threshold. Nevertheless, given the limited number of loci, the power to detect selection was limited. Thus, none of the 12 microsatellite loci showed statistical evidence of departure from neutral expectations in this analysis.

Possible null alleles were indicated only at HLJ338 and are unlikely to explain this general pattern because null alleles typically generate heterozygote deficits. Moreover, exclusion of HLJ338 produced only minor changes in the sensitivity analyses. Although STRUCTURE revealed admixed ancestry patterns, the available analyses do not establish whether admixture, gene flow, sampling effects, or other processes caused the observed heterozygote excess. The analyses confirmed the presence of heterozygote excess but did not identify its underlying biological or sampling-related cause.

The number of private alleles (Pa) varied among populations, with Shkodra exhibiting the highest value (20), whereas the Tapiza hatchery stock exhibited the lowest number (7). Several loci showed departures from Hardy–Weinberg equilibrium, as detailed in Table 3. However, the present analyses do not identify the processes responsible for these deviations. Detailed locus-specific results are provided in Table S2.

According to the AMOVA analysis (Table 4), the greatest proportion of genetic variation was partitioned within individuals (97%), whereas only 3% was attributed to differences among populations. Variation among individuals within populations was negligible, indicating no additional structuring at this level. Despite the low proportion of among-population variation, the fixation index was significant (F_ST_ = 0.0348, *p* < 0.001), demonstrating the presence of weak but significant genetic differentiation.

A sensitivity AMOVA excluding HLJ338 produced results virtually identical to those obtained with the complete 12-locus dataset. The proportion of variation among populations remained 3%, and the global fixation index remained unchanged and statistically significant (F_ST_ = 0.034, *p* = 0.001; Table S3). Thus, exclusion of the locus showing evidence of potential null alleles did not affect the inferred magnitude of population differentiation.

### 3.3. Population Genetic Structure

**Figure 2:**
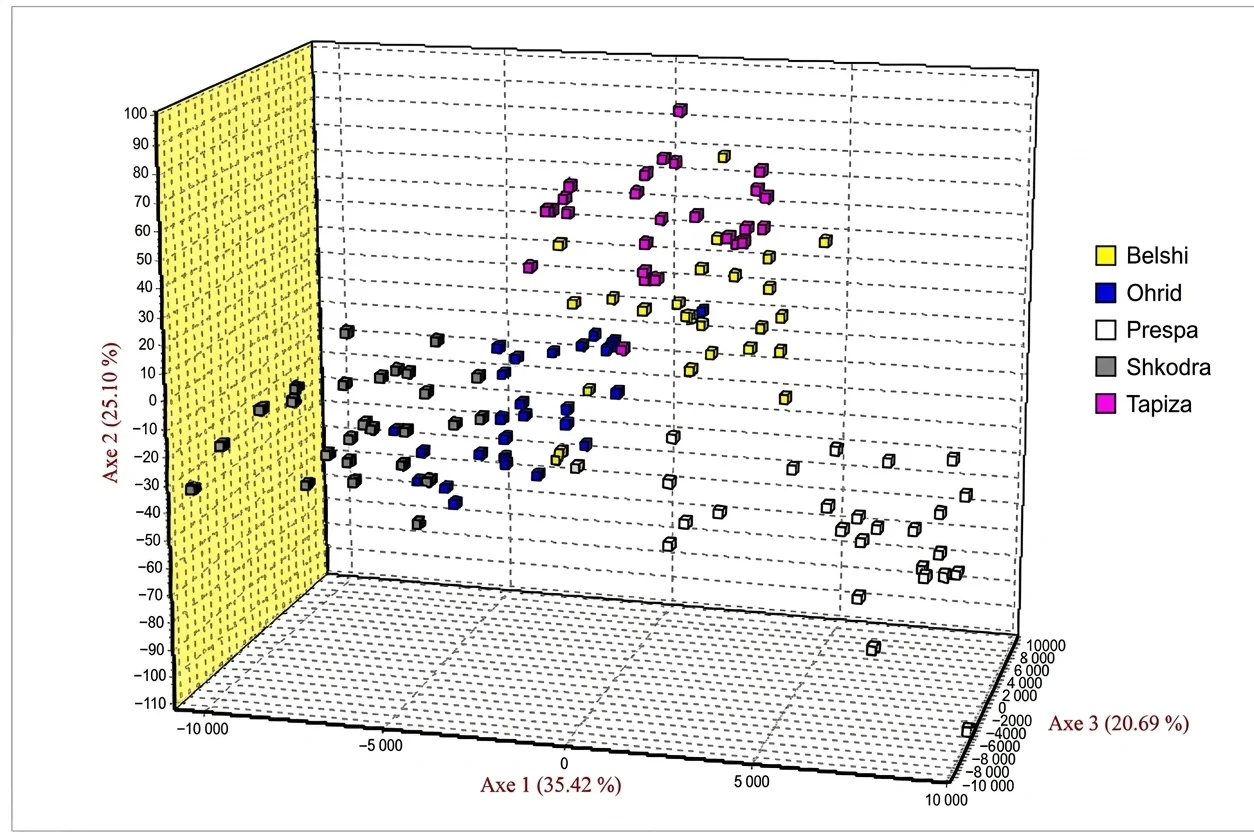
Factorial Correspondence Analysis (FCA) illustrating the distribution of individuals from five carp populations based on microsatellite data.

The Factorial Correspondence Analysis (FCA, Figure 2) revealed slight genetic differentiation among the studied carp populations. The first three axes explained 35.42%, 22.52%, and 20.69%, respectively, accounting for 78.63% of the total genetic variation. Along the first axis, the Prespa population tended to separate from the Ohrid and Shkodra populations, which largely overlapped, indicating a high degree of genetic similarity between these two lakes. Belshi and Tapiza occupied central positions along the first axis and showed partial overlap with each other, suggesting the occurrence of genetic affinity. The second and third axes provided limited additional differentiation, with partial clustering and overlap among populations. In particular, moderate levels of genetic affinity occurred between two groups of population: Ohrid and Shkodra, and Belshi and Tapiza. Notably, along the first three axes, the Prespa population remained separated from the other four populations.

The Principal Coordinate Analysis (PCoA, Figure S1) could be considered partially consistent with the FCA results, although axis 1 explained 6.31% of the total genetic variation, whereas axes 2 and 3 explained 4.84% and 4.05%, respectively. Although slight tendencies for separation among some populations were observed, particularly between the Ohrid population and the most divergent individuals from Prespa and Tapiza, the general overlap among populations indicated weak genetic structuring. However, the three principal coordinates together explained 15.20% of the total genetic variation; therefore, these patterns should be interpreted with caution.

Pairwise linkage disequilibrium analysis produced valid contingency tables for 311 of the 330 possible locus-pair-by-population comparisons. At the nominal significance level of *p* < 0.05, significant genotypic associations were detected in 36 comparisons: five in Belshi, four in Ohrid, six in Prespa, six in Shkodra, and 15 in Tapiza. Fisher’s combined tests across populations identified significant associations for 11 of the 66 locus pairs. After Bonferroni correction, the strongest combined evidence of linkage disequilibrium was observed for HLJ044–HLJ058 (*p* < 1.65 × 10^−10^) and HLJ873–HLJ338 (*p* < 2.23 × 10^−7^). No locus pair showed significant disequilibrium in all five populations, indicating that statistically significant LD was restricted to a small number of population-specific combinations rather than being widespread across the complete marker panel.

The limited and predominantly population-specific distribution of significant LD suggests that strong multilocus disequilibrium was not widespread across the marker panel. The physical linkage relationships among the microsatellite loci were not available and could not be evaluated directly.

**Figure 3:**
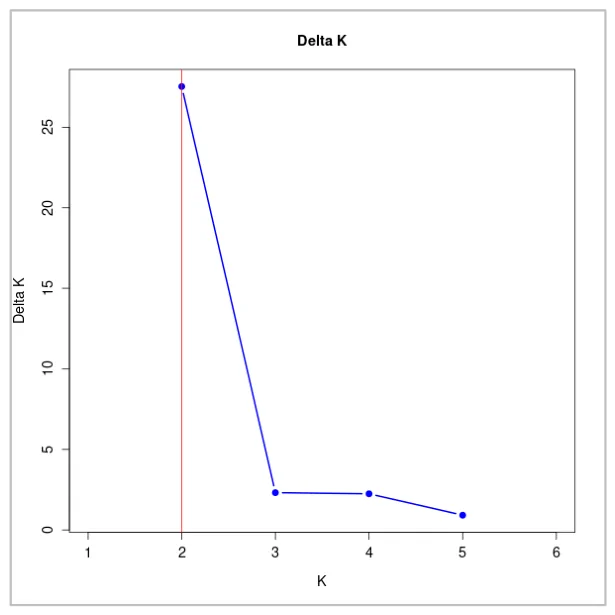
Values of the Evanno ΔK statistic for K = 2–5. The pronounced maximum at K = 2 indicates the strongest uppermost level of genetic structure. ΔK cannot be calculated for K = 1 or for the highest tested K. X-axis (K): the assumed number of genetic clusters tested in the STRUCTURE analyses. Y-axis (ΔK): the Evanno statistic, which measures the rate of change in the log probability of the data between successive K values. Higher ΔK values indicate stronger statistical support for that level of genetic structure.

**Figure 4:**
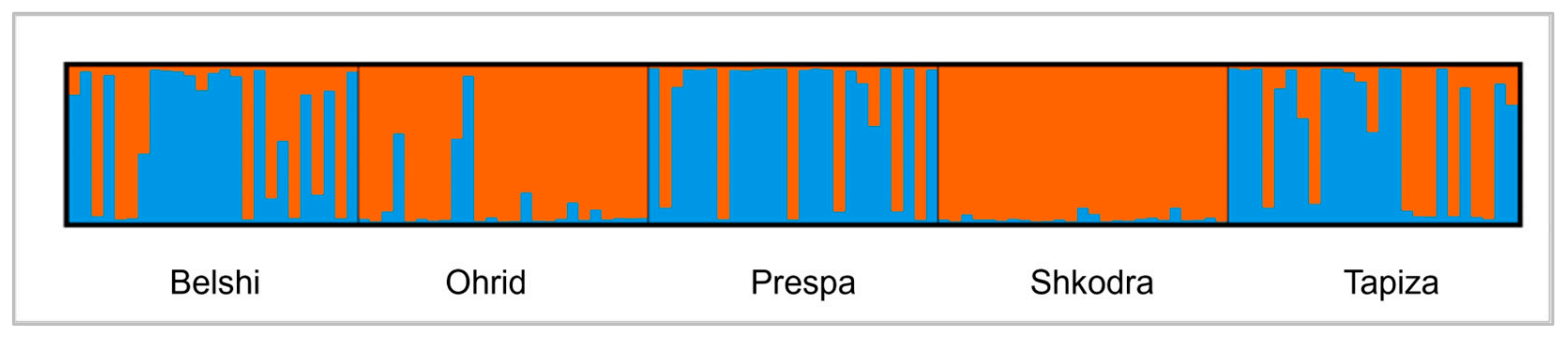
Bayesian clustering of carp populations inferred using STRUCTURE at K = 2. Each vertical bar represents an individual, and colors indicate the proportion of membership to each inferred genetic cluster. Individuals are grouped by population, showing partial differentiation and a high level of admixture.

Population structure was evaluated using Bayesian clustering for K values ranging from 1 to 6. The Evanno method identified a pronounced maximum at K = 2 (ΔK = 27.52), whereas substantially lower values were obtained for K = 3 (ΔK = 2.31), K = 4 (ΔK = 2.24), and K = 5 (ΔK = 0.92; Figure 3). Thus, K = 2 represented the strongest uppermost level of genetic structure. This solution was also highly consistent across replicate runs, with all 10 runs forming a single CLUMPAK major mode and a mean similarity score of 0.995. Accordingly, K = 2 was retained as the principal clustering solution presented in Figure 4.

Complementary criteria indicated plausible finer-scale structure at higher K values. The highest overall mean lnP(D) was observed at K = 3. The Puechmaille estimators unanimously supported K = 2 at membership thresholds of 0.6 and 0.7, whereas three of the four estimators supported K = 3 at the more permissive threshold of 0.5; only MaxMedK supported K = 4 at this threshold. CLUMPAK showed that all 10 runs at K = 3 formed a single major mode with a mean similarity score of 0.983. At K = 4, nine of the ten runs formed a consistent major mode with a mean similarity score of 0.923, whereas one divergent, low likelihood run formed a separate minor mode. The K = 3 and K = 4 major-mode solutions are therefore presented in Figure S2 as complementary finer-scale representations.

At K = 2, individuals displayed varying proportional membership in both inferred clusters, although their relative contributions differed among sampling populations (Figure 4). Considerable shared ancestry was also evident in the K = 3 and K = 4 solutions (Figure S2). Overall, these results indicate weak but detectable population structure accompanied by substantial admixture, consistent with the low but statistically significant global F_ST_.

GeneClass2 self-assignment correctly assigned 80 of 125 individuals (64.0%) to their sampling populations. Assignment success was highest in Shkodra (84%), followed by Prespa (68%), Belshi (64%), Ohrid (60%), and Tapiza (44%). After excluding HLJ338, 82 of 125 individuals (65.6%) were assigned to their sampling populations. Thus, removal of the locus showing evidence of possible null alleles changed overall self-assignment success by only 1.6 percentage points.

The primary L_home_ analysis identified five individuals (4.0%) as potential first-generation migrants at *p* < 0.01. All five remained below this threshold after excluding HLJ338, and one additional individual was detected in the 11-locus analysis. The complementary L_home_/L_max_⁡ analysis identified nine candidates with 12 loci and six after excluding HLJ338, with six candidates shared between the two datasets. Thus, exclusion of HLJ338 did not alter the overall evidence for a limited number of potential migrants, although the classification of some individuals was locus-sensitive. Because no correction for multiple testing was applied, these results were interpreted as exploratory and do not provide direct evidence of contemporary gene flow.

### 3.4. Pairwise Genetic Differentiation and Phylogenetic Relationships

Pairwise genetic differentiation and gene flow among carp populations are presented in Table 5. Pairwise F_ST_ values were consistently low, ranging from 0.0146 (Ohrid–Shkodra) to 0.0506 (Prespa–Shkodra), indicating weak genetic differentiation among populations. In accordance with FCA results (Figure 2), the lowest differentiation was observed between Ohrid and Shkodra, whereas Prespa and Shkodra exhibited the highest, although still relatively low, level of differentiation.

**Figure 5:**
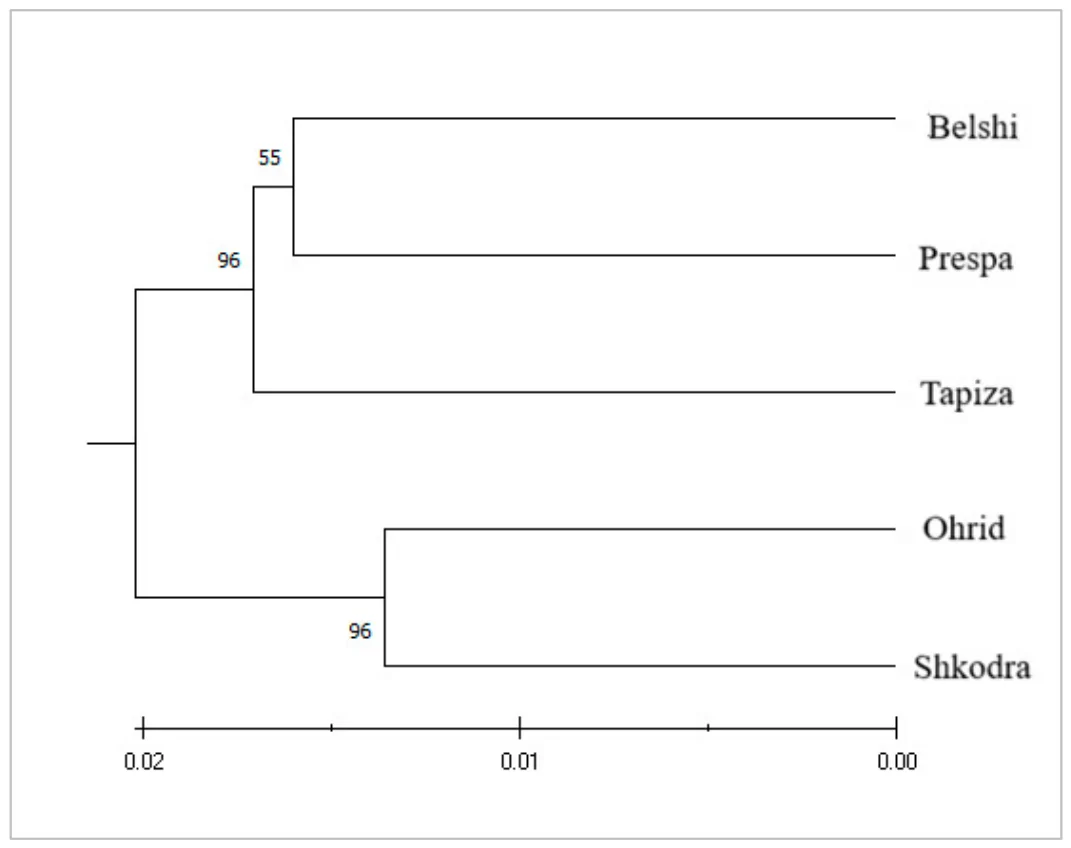
Neighbor-Joining (NJ) dendrogram constructed from Nei’s genetic distance illustrating the genetic relationships among carp populations. Branch lengths are proportional to genetic distance, with shorter branches indicating closer genetic similarity.

Estimates of gene flow (Nm) ranged from 4.69 to 16.83, with the highest Nm value observed between Ohrid and Shkodra (Nm = 16.83), consistent with their low F_ST_ value and high genetic similarity. Additionally, even the lowest estimates exceeded the threshold of Nm = 1. Population-level genetic relationships were examined through the implementation of a Neighbor-Joining (NJ) phylogenetic tree derived from Nei’s genetic distance (Figure 5).

Two major clusters were identified: the first, strongly supported by bootstrap values, grouped the Ohrid and Shkodra populations. The second cluster included Belshi, Prespa and Tapiza. Within this group, Belshi and Prespa formed a sub-cluster with weak bootstrap support, whereas Tapiza formed a distinct branch with stronger bootstrap support.

Accordingly, even considering that results should be interpreted cautiously, the Neighbour-Joining phylogenetic tree based on individual genotypes (Figure S3) showed a broad distribution of individuals from the five populations, with no clear grouping according to the sampling locality. Nevertheless, some localised clustering patterns were observed. Specifically, several individuals from Ohrid and Shkodra occurred in neighbouring branches. Likewise, a few Prespa individuals formed small population-specific genetic clusters. In contrast, individuals from Belshi and Tapiza were widely distributed across the phylogenetic tree and did not form exclusive clusters.

### 3.5. Assessment of Recent Bottlenecks and Effective Population Size Dynamics

Table 6 summarizes multiple statistical indicators used to detect recent bottleneck events under different mutation models. Under the two-phase mutation model (TPM), which is frequently considered a realistic mutation model for microsatellite data, both the sign test and the Wilcoxon signed-rank test were non-significant for all populations (*p* > 0.05). The TPM-based analyses detected no significant departure from mutation–drift equilibrium and provided no evidence of a recent severe bottleneck in the sampled populations.

Some significant values were observed under the infinite allele model (IAM) and the stepwise mutation model (SMM), particularly for Ohrid (IAM, *p* = 0.019) and for Belshi, Ohrid, and Tapiza under SMM (*p* < 0.05). The mode-shift indicator further supports the absence of bottlenecks, as all populations exhibited a normal L-shaped allele frequency distribution, which is consistent with the absence of a pronounced recent mode shift. The analysis did not provide consistent evidence across mutation models for a recent severe bottleneck.

Using the complete 12-locus panel and a minimum allele-frequency threshold of 0.02, NeEstimator produced finite LD-based estimates of contemporary effective population size for all populations. Tapiza had the lowest point estimate and Shkodra the highest, although confidence intervals were generally broad (Table S4).

The sensitivity analyses indicated some variation in Ne estimates among marker panels, particularly for populations with higher point estimates. However, Tapiza consistently retained the lowest estimate (Table S4). The complete 12-locus panel was therefore treated as the primary analysis, while the reduced panels were used as sensitivity checks, and the results were interpreted cautiously.

## 4. Discussion

This study characterizes patterns of genetic variation and population-level structuring in *Cyprinus carpio* from Albania, inferred by microsatellites, analysing five populations from the following sampling sites: the natural lakes of Ohrid and Shkodra, the semi-managed environments of Prespa and Belshi, and the hatchery stock of Tapiza.

Compared with previous investigations conducted in Albania, this study broadens the characterization of genetic diversity in common carp by focusing on two key aspects. First, the use of an expanded panel of polymorphic microsatellite loci allowed a fine detailed characterization of genetic variability, improving the resolution of population structure and genetic relationships among Albanian populations of *Cyprinus carpio*. Indeed, while earlier studies laid important foundations using the marker panels available at the time (e.g., [23,24]), the expanded panel used in this study provides higher resolution to detect finer-scale patterns of genetic differentiation.

Second, the inclusion of the Belshi population extends the geographic and ecological representation of Albanian freshwater systems by incorporating a small, semi-managed lake environment that has been poorly investigated in previous genetic surveys.

All populations exhibited substantial within-population genetic diversity, as indicated by their high levels of observed heterozygosity and allelic richness. These findings suggest that inbreeding has had only a limited impact on the genetic variability of the populations here analysed. Moreover, the Tapiza hatchery sample did not differ significantly in genetic diversity from the populations living in natural environments. This suggests that the hatchery population may have not undergone the marked loss of genetic variation often associated with long-term captive breeding or founder effects prompted by a limited number of broodstocks. These findings are consistent with the general pattern reported in other European common carp populations. Similar results have been recently described in populations from Poland and eastern Hungary, where microsatellites analyses revealed observed heterozygosity slightly exceeding expected heterozygosity, and heterozygote excess in several strains [7,17]. However, it should also be taken into consideration that other studies on both domestic and feral common carps from different regions of Hungary and Croatia reported heterozygote deficiency, likely reflecting differences in management intensity and breeding strategies (e.g., [21,22]).

Although all Albanian populations displayed a general high genetic diversity, some differences regarding the distribution of genetic variation within populations were evident. First, the population from the Shkodra natural lake exhibited the highest allelic richness and the greatest number of private alleles despite a long history of stocking practices [9,10,54]. The maintenance of high genetic diversity suggests that the natural and environmental characteristics of the lake may have buffered the effects of anthropogenic pressures on the genetic composition of the population, highlighting the potential importance of the Lake Shkodra population as a valuable reservoir of genetic diversity within Albania.

On the other hand, the Tapiza hatchery stock, despite maintaining relevant levels of heterozygosity, showed the lowest allelic richness and the fewest number of private alleles. This pattern has been reported in other hatchery populations, where founder effects and broodstock management may have reduced allelic diversity while preserving heterozygosity through controlled breeding and the use of a sufficiently large broodstock [8].

At the same time, our results revealed weak genetic structure among Albanian common carp populations, despite the high levels of within-population genetic diversity, with relatively high estimates of gene flow and extensive genetic admixture among populations inhabiting different natural and managed freshwater ecosystems. Since the freshwater systems analysed in this study are largely isolated from one another, with the partial exception of Lakes Ohrid and Prespa, this shows a limited karstic connection [55]. However, it should be taken into consideration that the observed genetic connectivity and weak genetic differentiation is unlikely to be explained by natural dispersal. Although these patterns may resemble those expected under a gene flow model, such connectivity is likely indirect given the lack of geographic connections among most studied sites. For these reasons, estimates of gene flow should not be interpreted as direct measures of contemporary migration, as they may also reflect underlying historical demographic processes, shared ancestry, population size, mutation, and the assumptions of the underlying model. Therefore, the observed genetic similarity among geographically separated populations may result from the repeated introduction of fingerlings, potentially originating from shared or genetically similar and highly variable broodstock, or from historical stocking events involving fish derived from the same genetic source (for example, see [56]). Such repeated translocations have been reported to promote genetic homogenization among geographically isolated freshwater populations by reducing genetic differentiation through the widespread use of common hatchery stocks [57]. A similar pattern was reported by Thai et al. [58], who investigated the genetic diversity and population structure of common carp populations in Vietnam. They observed limited genetic differentiation among populations from different geographic regions and suggested that the widespread exchange of cultured stocks and the use of common hatchery sources contributed to the homogenization of genetic patterns among populations. This trend of inter-population genetic homogeneity among *Cyprinus carpio* populations detected at the regional scale (e.g., [58]; present study) appears to extend to the continental scale. Indeed, Kohlmann et al. [16] investigated the genetic variability of common carp populations across Europe, Central Asia, and East/South-East Asia, evidencing that wild, domesticated and feral populations mostly shared genetic patterns, attributed to the extensive use of hatchery stocks, translocations, and exchanges of breeding material.

Bayesian clustering analysis identified two main genetic groups widespread across all populations. Although both groups were detected in all sampled populations, their relative frequencies differed among them. In particular, individuals from the two natural lakes, Ohrid and Shkodra, showed a clear predominance of only one of the two genetic groups, while populations of Belshi and Prespa, along with the Tapiza hatchery showed a more balanced contribution of both genetic lineages. This pattern of genetic admixture, potentially compatible with the effects of historical stocking practices and anthropogenic movements of fish, suggests also that the Ohrid and Shkodra populations, living in natural lakes, could have retained a more homogeneous genetic composition. Such a finding could be related to the potential greater ecological stability and larger spatial extent of these lake systems, which may support larger effective population sizes and consequently reduce the influence of genetic drift, favouring the long-term maintenance of a more homogeneous genetic composition [25,59]. Although, at the current stage of knowledge, the contribution of selective processes cannot be ruled out, given the evidence of extensive historical stocking and the use of hatchery-derived fish, the observed genetic patterns may be more consistent with demographic processes, including repeated introductions from genetically similar broodstock and disassortative mating system, than with selective sweeps [4,25].

This finding is consistent with the multivariate and phylogenetic analyses, in which *Cyprinus carpio* individuals from Lake Ohrid and Lake Shkodra clustered more closely together than those from the other populations, indicating a higher degree of genetic homogeneity. In contrast, in the managed environments of Belshi, Prespa, and Tapiza the historical influence of stocking practices and human-mediated translocations may have partly produced the increased genetic admixture observed. In this context, in a previous study, Polish breeding strains showed high genetic differentiation, likely reflecting restricted gene flow and breeding isolation [17].

Furthermore, the absence of clear bottleneck signatures found in the present study is consistent with the low genetic differentiation observed, suggesting that possible constant and repeated human-mediated introductions of *Cyprinus carpio* individuals potentially originating from a common genetic pool, together with local environmental conditions, may have minimized the effects of the genetic drift (i.e., the drift of allelic frequencies) on their genetic composition. Indeed, despite some differences among mutation models, neither the mode-shift analysis nor the estimates of effective population size provided evidence of recent severe reductions in population size. Therefore, the observed genetic pattern is consistent with long-term genetic connectivity which may have been partly mediated by historical processes associated with anthropogenic activities, particularly stocking and translocation programmes, potentially contributing to the observed genetic similarity among freshwater systems.

## 5. Conclusions

The results obtained in this study should be interpreted on the basis of historical information on inland fisheries in Albania reported in the literature, which indicates that, until the early 1990s, state-owned hatcheries played a central role in the production of common carp fingerlings for nationwide stocking programmes [9,10,60]. Historical records further indicate that Lakes Shkodra, Ohrid, and Prespa were annually stocked with common carp fingerlings produced from broodstock collected separately from each lake, indicating an explicit intention to preserve the genetic characteristics of the respective lake populations. Additional historical accounts indicate that hatchery production and restocking were conducted on a broad scale in Albania [61]. Notably, the Tapiza hatchery represented one of the main breeding centres, maintaining genetically improved common carp broodstock and supplying fingerlings for restocking natural lakes, reservoirs, and other freshwater systems throughout the country [54]. Such repeated translocations might have promoted genetic similarity among geographically separated populations, potentially increasing genetic admixture and reducing genetic differentiation over time. Based on this scenario, it can be hypothesized that the broodstock historically used for stocking programmes could have retained high levels of genetic variability. Therefore, repeated stocking activities may have introduced individuals originating from genetically similar or shared broodstock sources into multiple freshwater systems. Consequently, the present-day Albanian common carp populations may not represent fully distinct genetic units but rather form a partially mixed genetic system shaped by shared ancestry and historical processes, possibly resulting from stocking and translocation events. However, the available records do not provide complete information on the broodstock origin, hatchery source, and destination of every stocking batch. These records suggest substantial and recurrent human-mediated propagation and stocking, but they neither establish that the sampled populations originated from a single historical broodstock nor demonstrate that stocking was the principal cause of the genetic patterns observed in the present study. In this context, the different local environmental conditions may have favoured the persistence of part of the original genetic variation despite repeated introductions, thereby limiting the loss of genetic diversity and reducing the potential effects of genetic drift and inbreeding in both hatchery and recipient populations. This hypothesis is also consistent with the genetic homogenisation reported by Yesiltaş et al. [62] in Turkish *Cyprinus carpio* populations, where extensive stocking and translocation activities progressively reduced population differentiation despite the persistence of high genetic variability within populations.

Although historical sources documenting stocking practices in Albania are limited, this scenario represents a plausible explanation for the observed findings considering the available information reported in the literature so far. However, further studies based on a large number of individuals from a broader geographic coverage, including additional freshwater systems and reservoirs across Albania, together with a higher number of molecular markers and updated historical information on stocking and translocation practices in the country, will be necessary to further evaluate this hypothesis and better understand the processes underlying the observed genetic patterns.

For instance, genome-wide analyses using high-density SNP markers would be required to disentangle the roles of historical management practices, demographic history and adaptive evolution, potentially identifying polymorphisms associated with recent or population-specific selective processes. Such information may provide a basis for the development of genetic traceability tools that could support the identification and monitoring of broodstock, help distinguish genetic stocks, trace the origin of cultured individuals, and improve the management of stocking practices in Albania.

In this context, the potential contribution of historical stocking and translocation practices to the maintenance of genetic diversity across the study area should be considered. However, it should be taken into account that an excessive level of genetic connectivity may also lead to genetic homogenisation, potentially resulting in the loss of locally adapted gene pools.

In conclusion, future management programmes should integrate genetic monitoring into stocking practices to balance genetic homogeneity among sites and the maintenance of genetic variation, thus preventing the potential effects of genetic drift and inbreeding depression within populations. Hatchery management should also take into consideration the genetic variability of broodstock, which should ideally be high, to avoid unintended impacts on populations. Therefore, the maintenance of the high levels of genetic variation observed in the present study, probably shaped by both historical and stochastic processes, should represent a primary objective for the future management plans of common carp in Albania.

## Figures and Tables

**Table 1:** Microsatellite loci selected for the genetic analysis of *Cyprinus carpio*, with corresponding forward and reverse primer sequences, repetitive motifs, and annealing temperatures used for PCR amplification [20].

Locus	Primer Sequence (5′→3′)	Repetitive Motif	Annealing T. (°C)
HLJ398	F: CATTACTTGAACTATCATCCA R: TGTGCTGAGGATTATTGG	(CT)_16_	53
HLJ044	F: GTACAGCGTGACAGCATT R: AAGTTCATCGGTGTCCTC	(CA)_28_	
HLJ058	F: CAGATGGCAGACAGGTAA R: GAGCAAGTGAGGGAACAG	(CA)_21_	
HLJ896	F: ATCACCAGTACATTCACT R: CGTTTAGCAAAGGTTAGT	(CA)_36_	
HLJ049	F: GATTTGTGCTCCTCAACC R: CTGTCACTTCTCCTTCCA	(GT)_28_	54
HLJ055	F: GGTACAACGGGAACCACA R: TGATTGACAGGCAGTGGG	(CT)_11_, (CA)_29_	
HLJ380	F: AGGCAGACGAAAGGTAAA R: CTCGCTTCTGTAGGCATT	(CA)_34_	
HLJ392	F: GGCTACAAGGCAACACTG R: TGCGGTTAATGAGGTCTG	(CA)_22_	
HLJ806	F: GGTGTCAGGCTTTAGTCC R: CATCTGAGTTTTCTCCAAGT	(CA)_48_	48
HLJ817	F: GACGATCCAGCAGCAATG R: CTCTTCCTAAAGCCTCAAA	(GT)_22_	
HLJ873	F: AGTGTCGTTTATGCGTATCTT R: AGCTCGCCTACTCTTCTACT	(CA)_50_	
HLJ338	F: GAAGAATGGGTGAGTAAGA R: ACTAGGATTTGGAAGAGC	(AC)_58_	51

F: forward primer; R: reverse primer.

**Table 2:** Genetic diversity parameters and F-statistics across 12 microsatellite loci in five carp populations.

L398.	TNA	Ae	I	H_O_	H_E_	uHe	PIC	AR	HW	F_IS_	F_IT_	F_ST_
HLJ398	9	4.615	1.657	0.867	0.762	0.777	0.782	7.440	***	−0.119	−0.065	0.049 ***
HLJ044	20	6.117	2.020	1.000	0.821	0.838	0.859	12.025		−0.199	−0.133	0.055 ***
HLJ058	24	3.311	1.638	0.984	0.696	0.711	0.693	10.680	**	−0.396	−0.376	0.015 ***
HLJ896	18	5.932	2.028	0.976	0.827	0.844	0.843	11.397	***	−0.160	−0.132	0.025 ***
HLJ049	18	6.110	2.028	0.958	0.823	0.840	0.836	11.949	***	−0.144	−0.119	0.022 ***
HLJ055	18	5.689	1.968	0.947	0.819	0.837	0.848	10.762	*	−0.139	−0.086	0.047 ***
HLJ380	18	6.290	2.040	0.991	0.832	0.850	0.879	11.651	***	−0.171	−0.098	0.063 ***
HLJ392	12	3.448	1.469	0.951	0.695	0.710	0.687	7.855	***	−0.347	−0.311	0.027 ***
HLJ806	25	7.794	2.275	0.957	0.862	0.881	0.887	14.742	***	−0.091	−0.060	0.028 ***
HLJ817	4	2.047	0.738	0.976	0.511	0.521	0.393	2.422	***	−0.906	−0.906	0.000
HLJ873	23	6.523	2.131	0.992	0.845	0.862	0.868	13.640		−0.154	−0.119	0.031 ***
HLJ338	23	6.771	2.123	0.709	0.847	0.867	0.885	13.218	***	0.178	0.210	0.038 ***
**Average**	17.667	5.387	1.843	0.942	0.778	0.795	0.788	10.648		**−0.192**	**−0.150**	0.035 **

TNA: total number of alleles; A_e_: effective number of alleles; I: Shannon’s information index; H_O_: observed heterozygosity; H_E_: expected heterozygosity; uH_e_: unbiased expected heterozygosity; PIC: polymorphic information content; AR: allelic richness; HW: locus-level Hardy–Weinberg equilibrium test based on Fisher’s combined probability across the five populations (*: *p* < 0.05; **: *p* < 0.01; ***: *p* < 0.001); F_IS_: inbreeding coefficient within populations; F_IT_: total inbreeding coefficient; F_ST_: genetic * differentiation among populations.

**Table 3:** Genetic diversity parameters of five carp (*Cyprinus carpio*) populations based on microsatellite markers.

Pop	MNA	A_e_	I	H_O_	H_E_	uH_e_	AR	F_IS_	P_a_	D(HWE) ^1^	D(HWE) ^2^
Belshi	9.667	5.248	1.812	0.924	0.772	0.789	7.704	−0.175	11	7	1
Ohrid	10.667	5.632	1.897	0.909	0.786	0.803	8.294	−0.134	13	5	3
Prespa	9.5	4.697	1.78	0.940	0.764	0.78	7.714	−0.214	13	8	3
Shkodra	11.5	6.711	2.031	0.946	0.814	0.831	8.993	−0.142	20	5	1
Tapiza	8.583	4.648	1.694	0.993	0.756	0.771	6.884	−0.296	7	8	4
Average	9.983	5.387	1.843	0.942	0.778	0.795	8.767	−0.192			

MNA: mean number of alleles; A_e_: effective number of alleles; I: Shannon’s information index; H_O_: observed heterozygosity; H_e_: expected heterozygosity; uH_e_: unbiased expected heterozygosity; AR: allelic richness; F_IS_: inbreeding coefficient; P_a_: number of private alleles; D(HWE): number of loci deviating from Hardy–Weinberg equilibrium (^1^ Nominal deviations *p* < 0.05; ^2^ After Bonferroni *p* < 0.00083).

**Table 4:** Analysis of molecular variance (AMOVA) for five populations based on microsatellite markers.

Source of Variation	df	Sum of Squares (SS)	Mean Squares (MS)	Variance Components	Percentage of Variation (%)
Among populations	4	50.132	12.533	0.169	3%
Among individuals within populations	120	487.600	4.063	0.000	0%
Within individuals	125	688.000	5.504	5.504	97%
**Total**	249	1225.732	—	5.673	100%

**Table 5:** Pairwise genetic differentiation and gene flow among carp (*Cyprinus carpio*) populations. Values above the diagonal represent F_ST_ estimates based on Weir and Cockerham [35], while values below the diagonal represent gene flow (Nm) calculated according to Wright [38].

	Belshi	Ohrid	Prespa	Shkodra	Tapiza
**Belshi**	–	0.0265	0.0236	0.0361	0.0275
**Ohrid**	9.18	–	0.0438	0.0146	0.0416
**Prespa**	10.36	5.46	–	0.0506	0.0413
**Shkodra**	6.67	16.83	4.69	–	0.0416
**Tapiza**	8.86	5.76	5.81	5.75	–

**Table 6:** Bottleneck detection tests under different mutation models (IAM, TPM, SMM), Wilcoxon signed-rank test, and mode-shift indicator for five carp populations.

Detection Indicator	Belshi	Ohrid	Prespa	Shkodra	Tapiza
**Sign test (IAM,** ***p*****)**	0.088	0.019 *	0.248	0.231	0.213
**Sign test (TPM,** ***p*** **)**	0.321	0.532	0.465	0.505	0.367
**Sign test (SMM,** ***p*****)**	0.004 **	0.003 **	0.364	0.173	0.023 *
**Wilcoxon (IAM, two-tailed** ***p*****)**	0.003 **	0.006 **	0.042 *	0.003 **	0.042 *
**Wilcoxon (TPM, two-tailed** ***p*****)**	0.569	0.970	0.677	0.791	0.910
**Wilcoxon (SMM, two-tailed** ***p*****)**	0.005 **	0.002 **	0.569	0.233	0.034 *
**Mode-shift indicator**	Normal L-shaped	Normal L-shaped	Normal L-shaped	Normal L-shaped	Normal L-shaped

Significant values are indicated as * *p* < 0.05 and ** *p* < 0.01.

## Data Availability

The original contributions presented in this study are included in the article/Supplementary Material. Further inquiries can be directed to the corresponding author.

## Supplementary Materials

The following supporting information can be downloaded at https://www.mdpi.com/article/10.3390/life16091524/s1, Figure S1: Principal Coordinate Analysis (PCoA) showing genetic relationships among five carp populations based on microsatellite markers: (a) Axis 1 vs. Axis 2; (b) Axis 1 vs. Axis 3. Figure S2. Bayesian clustering of 125 common carp individuals at (a) K = 3 and (b) K = 4. The plots represent the major clustering modes identified by CLUMPAK. Each vertical bar represents one individual, and colours indicate proportional membership in the inferred clusters. Figure S3: Neighbor-joining dendrogram based on Nei’s standard genetic distance illustrating the genetic relationships among common carp individuals from five populations (Ohrid, Prespa, Shkodra, Belshi, and Tapiza). Branch support values are not shown because bootstrap support is not reported for individual-level neighbour-joining trees generated by POPULATIONS v.1.2.28. Table S1: Estimated null allele frequencies in 5 carp populations using 12 microsatellite loci. Table S2: Genetic diversity parameters across loci and populations based on microsatellite markers. Table S3. Sensitivity analysis of molecular variance (AMOVA) among five common carp populations after exclusion of HLJ338, the locus showing evidence of potential null alleles. Table S4. Sensitivity of linkage-disequilibrium-based estimates of contemporary effective population size (Ne) to microsatellite marker-panel composition.
