# Supplementary material for: Genetic Diversity and Population Structure of the Common Carp (Cyprinus carpio) in Albania Revealed by Microsatellite Markers
Source: Life (Basel). 2026 Sep 14;16(9):1524. doi: 10.3390/life16091524 (PMC13608679; doi:10.3390/life16091524)
Supplement: Supplementary file 1 [file life-16-01524-s001.zip › Figures S1-S2-S3 Captions.pdf]

Figure S1: Principal Coordinate Analysis (PCoA) showing genetic relationships among five carp populations based on microsatellite markers: a) Axis 1 vs. Axis 2; b) Axis 1 vs. Axis 3.

Figure S2. Bayesian clustering of 125 common carp individuals at a) K = 3 and b) K = 4. The plots represent the major clustering modes identified by CLUMPAK. Each vertical bar represents one individual, and colours indicate proportional membership in the inferred clusters.

Figure S3. Neighbor-joining dendrogram based on Nei's standard genetic distance illustrating the genetic relationships among common carp individuals from five populations (Ohrid, Prespa, Shkodra, Belshi, and Tapiza). Branch support values are not shown because bootstrap support is not reported for individual-level neighbour-joining trees generated by POPULATIONS v.1.2.28.
