# Supplementary material for: Genetic Diversity and Population Structure of the Common Carp (Cyprinus carpio) in Albania Revealed by Microsatellite Markers
Source: Life (Basel). 2026 Sep 14;16(9):1524. doi: 10.3390/life16091524 (PMC13608679; doi:10.3390/life16091524)
Supplement: Supplementary file 1 [file life-16-01524-s001.zip › Supplementary Tables.pdf]

Table S1. Estimated null allele frequencies in 5 carp populations using 12 microsatellite loci.

Locus	Belshi	Ohrid	Prespa	Shkodra	Tapiza
**HLJ398**	0.005	0.000	0.000	0.109	0.000
**HLJ044**	0.000	0.000	0.000	0.000	0.000
**HLJ058**	0.000	0.000	0.000	0.000	0.000
**HLJ896**	0.000	0.000	0.000	0.000	0.000
**HLJ049**	0.000	0.003	0.000	0.000	0.000
**HLJ055**	0.000	0.000	0.000	0.000	0.000
**HLJ380**	0.000	0.000	0.000	0.000	0.000
**HLJ392**	0.000	0.000	0.000	0.000	0.000
**HLJ806**	0.007	0.000	0.027	0.000	0.000
**HLJ817**	0.000	0.000	0.000	0.000	0.000
**HLJ873**	0.000	0.000	0.000	0.000	0.000
**HLJ338**	0.125	0.134	0.151	0.009	0.000

Table S2. Genetic diversity parameters across loci and populations based on microsatellite markers.

Pop		HLJ398	HLJ044	HLJ058	HLJ896	HLJ049	HLJ055	HLJ380	HLJ392	HLJ806	HLJ817	HLJ873	HLJ338
**Belshi**	**N**	24	25	25	25	23	22	23	23	24	25	25	22
	**Na**	7	11	10	11	10	10	9	7	15	3	13	10
	**Ae**	3.282	6.281	3.472	6.944	4.967	5.728	6.260	2.606	8.662	2.080	7.225	5.469
	**I**	1.495	2.075	1.654	2.130	1.864	1.981	1.960	1.227	2.432	0.777	2.216	1.936
	**Ho**	0.792	1.000	1.000	0.960	0.957	0.955	0.957	0.957	0.917	1.000	1.000	0.591
	**He**	0.695	0.841	0.712	0.856	0.799	0.825	0.840	0.616	0.885	0.519	0.862	0.817
	**uHe**	0.710	0.858	0.727	0.873	0.816	0.845	0.859	0.630	0.903	0.530	0.879	0.836
	**F**	-0.139	-0.189	-0.404	-0.121	-0.198	-0.156	-0.138	-0.552	-0.036	-0.926	-0.161	0.277
**Ohrid**	**N**	25	25	25	24	24	20	23	25	20	25	25	19
	**Na**	9	12	11	15	11	9	11	6	14	2	15	13
	**Ae**	6.345	7.062	3.094	6.982	6.031	6.250	5.845	2.660	8.696	1.997	6.345	6.278
	**I**	1.989	2.167	1.647	2.270	2.028	1.968	2.055	1.252	2.363	0.692	2.198	2.140
	**Ho**	0.920	1.000	0.920	1.000	0.875	0.900	1.000	0.840	0.950	0.960	0.960	0.579
	**He**	0.842	0.858	0.677	0.857	0.834	0.840	0.829	0.624	0.885	0.499	0.842	0.841
	**uHe**	0.860	0.876	0.691	0.875	0.852	0.862	0.847	0.637	0.908	0.509	0.860	0.863

	**F**	-0.092	-0.165	-0.359	-0.167	-0.049	-0.071	-0.206	-0.346	-0.073	-0.923	-0.140	0.311
**Prespa**	**N**	24	25	25	25	24	25	25	25	25	25	25	23
	**Na**	5	8	14	12	10	11	11	6	14	3	11	9
	**Ae**	3.056	4.579	3.582	5.841	5.260	4.480	5.981	3.521	6.410	2.163	5.924	5.568
	**I**	1.322	1.760	1.873	2.103	1.925	1.907	2.030	1.449	2.222	0.833	2.031	1.910
	**Ho**	0.958	1.000	1.000	1.000	0.958	0.920	1.000	1.000	0.920	0.960	1.000	0.565
	**He**	0.673	0.782	0.721	0.829	0.810	0.777	0.833	0.716	0.844	0.538	0.831	0.820
	**uHe**	0.687	0.798	0.736	0.846	0.827	0.793	0.850	0.731	0.861	0.549	0.848	0.839
	**F**	-0.425	-0.279	-0.387	-0.207	-0.183	-0.184	-0.201	-0.397	-0.090	-0.786	-0.203	0.311
**Shkodra**	**N**	24	25	25	25	25	25	25	25	24	25	25	20
	**Na**	8	16	11	10	15	10	15	10	15	2	12	14
	**Ae**	6.295	8.865	3.444	5.365	9.921	4.717	8.993	4.699	10.378	1.997	7.440	8.421
	**I**	1.930	2.442	1.710	1.947	2.478	1.842	2.398	1.831	2.504	0.692	2.243	2.352
	**Ho**	0.667	1.000	1.000	0.960	1.000	0.960	1.000	0.960	1.000	0.960	1.000	0.850
	**He**	0.841	0.887	0.710	0.814	0.899	0.788	0.889	0.787	0.904	0.499	0.866	0.881
	**uHe**	0.859	0.905	0.724	0.830	0.918	0.804	0.907	0.803	0.923	0.509	0.883	0.904
	**F**	0.207	-0.127	-0.409	-0.180	-0.112	-0.218	-0.125	-0.220	-0.107	-0.923	-0.155	0.035
**Tapiza**	**N**	25	25	25	25	25	25	25	25	25	25	25	25
	**Na**	6	9	6	8	11	11	9	8	10	2	10	13
	**Ae**	4.098	3.799	2.962	4.529	4.371	7.267	4.371	3.754	4.826	2.000	5.682	8.117
	**I**	1.547	1.656	1.308	1.692	1.845	2.142	1.755	1.585	1.856	0.693	1.969	2.279
	**Ho**	1.000	1.000	1.000	0.960	1.000	1.000	1.000	1.000	1.000	1.000	1.000	0.960
	**He**	0.756	0.737	0.662	0.779	0.771	0.862	0.771	0.734	0.793	0.500	0.824	0.877
	**uHe**	0.771	0.752	0.676	0.795	0.787	0.880	0.787	0.749	0.809	0.510	0.841	0.895
	**F**	-0.323	-0.357	-0.510	-0.232	-0.297	-0.160	-0.297	-0.363	-0.261	-1.000	-0.214	-0.095
**Whole population**	**N**	24.400	25.000	25.000	24.800	24.200	23.400	24.200	24.600	23.600	25.000	25.000	21.800
	**Na**	7.000	11.200	10.400	11.200	11.400	10.200	11.000	7.400	13.600	2.400	12.200	11.800
	**Ae**	4.615	6.117	3.311	5.932	6.110	5.689	6.290	3.448	7.794	2.047	6.523	6.771
	**I**	1.657	2.020	1.638	2.028	2.028	1.968	2.040	1.469	2.275	0.738	2.131	2.123
	**Ho**	0.867	1.000	0.984	0.976	0.958	0.947	0.991	0.951	0.957	0.976	0.992	0.709
	**He**	0.762	0.821	0.696	0.827	0.823	0.819	0.832	0.695	0.862	0.511	0.845	0.847
	**uHe**	0.777	0.838	0.711	0.844	0.840	0.837	0.850	0.710	0.881	0.521	0.862	0.867
	**F**	-0.154	-0.224	-0.414	-0.181	-0.168	-0.158	-0.193	-0.376	-0.114	-0.912	-0.174	0.168

Table S3. Sensitivity analysis of molecular variance (AMOVA) among five common carp populations after exclusion of HLJ338, the locus showing evidence of potential null alleles.

**Source of variation**	**df**	**Sum of squares (SS)**	**Mean squares (MS)**	**Variance components**	**Percentage of variation (%)**
Among populations	4	44.708	11.177	0.154	3%
Among individuals within populations	120	419.180	3.493	0.000	0%
Within individuals	125	649.000	5.192	5.192	97%
**Total**	249	1112.888		5.346	100%

Table S4. Sensitivity of linkage-disequilibrium-based estimates of contemporary effective population size (Ne) to microsatellite marker-panel composition.

**Population**	**12 loci: Ne (parametric 95% CI)**	**Without HLJ338**	**Without HLJ338 and HLJ044**	**Without HLJ338 and HLJ058**
Belshi	98.9 (57.1–303.6)	84.6	122.0	118.1
Ohrid	78.2 (50.0–163.0)	87.5	119.9	94.3
Prespa	58.6 (40.7–98.3)	58.2	71.3	36.5
Shkodra	149.2 (80.8–711.8)	108.7	205.7	150.4
Tapiza	27.8 (21.8–36.8)	27.6	35.7	27.0
